# Maternal Serum Delta-Like 1 and Nesfatin-1 Levels in Gestational Diabetes Mellitus: A Prospective Case-Control Study

**DOI:** 10.7759/cureus.17001

**Published:** 2021-08-08

**Authors:** Melike Demir Çaltekin, Ayşen Caniklioğlu

**Affiliations:** 1 Obstetrics and Gynecology, Yozgat Bozok University, Faculty of Medicine, Yozgat, TUR; 2 Biochemistry, Yozgat Bozok University, Faculty of Medicine, Yozgat, TUR

**Keywords:** adipokine, delta-like 1 (dlk1), gestational diabetes mellitus, insulin resistance, nesfatin-1

## Abstract

Objective

Delta-like 1 (DLK1) and nesfatin-1 are adipokines that have been shown to affect glucose metabolism. We aimed to search serum DLK1 and nesfatin-1 concentrations at 24-28 weeks of pregnancy in women newly defined with gestational diabetes mellitus (GDM) and investigate the relationship of these adipokines with various metabolic parameters.

Methods

Serum levels of DLK1 and nesfatin-1 were evaluated in 44 women with GDM, and in 40 healthy pregnant women by enzyme-linked immunosorbent assay (ELISA) kits. While performing oral glucose tolerance test (OGTT) for GDM diagnosis at 24-28 weeks of pregnancy, homeostasis model assessment of insulin resistance (HOMA-IR), lipid profiles, glycosylated hemoglobin (HbA1c) were also measured.

Results

Maternal serum DLK1 and nesfatin-1 concentrations were found lower in pregnant women with GDM compared with healthy pregnant women (418.4±282.6 vs. 586.7±303 ng/L, p=0.002; 12.2±7.6 vs. 26.7±16.4 ng/ml, p<0.001, respectively). Maternal serum DLK1 levels correlated positively with HOMA-IR and fasting insulin (r=0.395, p=0.008; r=0.374, p=0.012, respectively).

Conclusion

We determined that DLK1 and nesfatin-1 levels were lower in GDM. Based on this study, it may be considered that DLK1 could be culpable for metabolic disorders in GDM.

## Introduction

Gestational diabetes mellitus (GDM) is the development of glucose intolerance that begins during pregnancy or diagnosed for the first time during pregnancy and is one of the most common complications of pregnancy. Albeit the incidence of GDM varies depending on the type of the selected test and the studied population, it occurs in 5% to 20% of all pregnancies [1]. Its incidence is rising across the world in parallel with the increment in the incidence of obesity and diabetes mellitus [2]. GDM causes short and long-term complications in both mother and fetus exposed to hyperglycemia. Pregnant women with GDM are at an increased risk of metabolic diseases like obesity, hypertension, dyslipidemia, cardiovascular disease, insulin resistance, and the risk of developing type 2 diabetes mellitus (T2DM) increases at least seven-fold among these women with GDM [3,4]. Besides, it might lead to the transmission of cardio-metabolic diseases to future generations via the children of GDM mothers, due to the incremented risk of developing diabetes and obesity in the later stages of their lives [5]. For these reasons, it is crucial to shed light on the pathogenesis of GDM for the prevention and treatment of the disease.

Maternal adiposity is a substantial alterable risk factor for GDM development [4]. It is also accountable for the synthesis and secretion of bioactive molecules, namely adipokines, apart from the lipid storage function of adipose tissue. Recent research indicates that adipose tissue dysfunction, which is characterized by abnormal adipokine production, might play an important role in the pathophysiology of GDM [6,7].

Delta-like 1 (DLK1), which is also called a fetal antigen (FA1) or preadipocyte factor 1 (Pref-1) is an epidermal growth factor repeat-containing transmembrane protein, considerably akin to the Notch/Delta/Serrate family [8]. DLK1 disappears during adipogenesis, and is not detected in adipocytes, whereas it is highly expressed in preadipocytes; hence, it is considered a preadipocyte marker [9]. It is expressed in the long arm of chromosome 14 in humans, and defects in this region have been associated with obesity, diathesis, insulin resistance, and impaired glucose tolerance [10]. It is well-known that it plays a role in glucose hemostasis; however, the results regarding its impact are contradictory [11]. Furthermore, it is put forward that intrauterine hyperglycemia leads to abnormal placental DLK1 expression due to DNA methylation among next-generation mice [12]. In this regard, DLK1 might play an important role in the pathophysiology of obesity and T2DM, which are common in the offspring of GDM mothers in the later stages of their life.

Nesfatin-1, on the other hand, is a nucleobindin-2 (NUCB2) originated adipokine that plays a role in metabolic processes including glucose homeostasis, and has a glucose-dependent insulinotropic effect. Its expression has been demonstrated in the central nervous system, gastro-endocrine cells, adipocytes, and pancreatic beta cells [13]. It has been revealed that fasting plasma levels of nesfatin-1 are decreased among patients with T2DM and polycystic ovary syndrome (PCOS). Nesfatin-1 levels were also assessed in GDM, which has similar pathophysiological characteristics with T2DM and PCOS, and conflicting results have been revealed in the literature [1,14-17].

Data on both DLK1 and nesfatin-1 levels and roles in GDM are still unclear. In our study, we aimed to assess the concentrations of both adipokines among pregnant women with GDM, concurrently with anthropometric and metabolic parameters.

## Materials and methods

This case-control study was conducted between January and August 2020 at the obstetrics and gynecology department of Yozgat Bozok University, Faculty of Medicine upon obtaining the approval of the local Ethics Committee (protocol number: 2017-KAEK-189_2019.12.11_14). The ethical principles of the Declaration of Helsinki were adhered to throughout the study and written informed consent was obtained from all participants.

Study population

In this study, 44 outpatient pregnant women aged between 18 years and 39 years who were diagnosed with GDM were included in the study group, and 40 healthy pregnant women who were matched for age, pre-pregnancy BMI, and gestational age were included in the control group. Gestational age was calculated by menstrual dating and first-trimester ultrasonography. Patients diagnosed with type 1 and type 2 diabetes, hypertension, multiple pregnancies, chronic heart, kidney and liver disease, smoking, alcohol use, drug use that may affect insulin metabolism, and patients with obstetric complications other than GDM were excluded from the study. A two-step approach was used for the diagnosis of GDM in our study, as it was also routinely preferred in our clinic. A 50 g of oral glucose challenge test was applied on all pregnant women between the 24th and 28th gestational weeks for gestational diabetes mellitus screening. A 100 g of oral glucose tolerance test (OGTT) was performed on the pregnant women whose serum glucose (SG) level in the first hour was determined to be at 140 mg/dl and over, following a diet rich in carbohydrates for three days and thereafter 12-hour fasting. First, second, and third hour SG levels were assessed. The pregnant women were diagnosed with GDM, in the event of having two of the following criteria: fasting SG>95 mg/dl, first hour SG>180 mg/dl, second hour SG>155 mg/dl, and third hour SG>140 mg/dl [18].

Height and weight measurements of all women were recorded, and body mass index (BMI) was calculated as weight/height^2^ (kg/m^2^). Height was measured by using standard wall-mounted tape in a standing position. Weight was measured by using a digital scale. The weight was taken in light clothes and the height without shoes. BMI was recalculated at the time of blood sampling. In addition, maternal age (year), gravidity, parity, gestational age at blood sampling (days), weight gain from the beginning of pregnancy until blood sampling day, gestational age at delivery (days), neonatal birth weight (g), type of delivery, neonatal gender, and neonatal Appearance, Pulse, Grimace, Activity, and Respiration (APGAR) score (1 min and 5 min) were recorded.

Laboratory measurements

Venous blood samples were collected between 8:00 am and 9:00 am after fasting for at least eight hours. All analyzes were studied on the same day, except nesfatin-1 and DLK1. Fasting glucose (FG), total cholesterol (TC), high-density lipoprotein cholesterol (HDL-C), triglyceride (TG), and HbA1c levels were analyzed on Roche COBAS 6000 c501 (Mannheim, Germany: Roche Diagnostics) autoanalyzer. Low-density lipoprotein cholesterol (LDL-C) was calculated using the Friedewald formula when the triglyceride level was less than 400 mg/dL.

Fasting serum insulin (FSI) was assessed by the electrochemiluminescence immunoassay (ECLIA) on Roche COBAS 6000 e601 (Mannheim, Germany: Roche Diagnostics) autoanalyzer. Insulin resistance was determined using the homeostatic model assessment insulin resistance index (HOMA-IR): fasting serum glucose (mg/dL) × fasting serum insulin (mU/mL)/405 [19].

Serum samples for nesfatin-1 and DLK1 were centrifuged for 10 min at 3000 rpm, after which the supernatant was quickly removed and kept frozen at −80°C until the assays were performed by an expert who was unaware of the patient's condition. The concentrations of serum nesfatin-1 and DLK1 were measured using commercially available ELISA kits (Shanghai, China: Bioassay Technology Laboratory), according to the manufacturer's protocols.

Statistical analysis

Statistical package program SPSS 20 (Armonk, NY: IBM Corp.) was used to interpret the data. Data were expressed as mean ± SD. Continuous variables were examined using Kolmogorov-Smirnov to determine whether or not they are normally distributed. The Mann-Whitney U-test was used for non-parametric numerical data and Student's t-test was used for parametric numerical data for pairwise comparison. Relationships between categorical variables were examined by the chi-square test. Bivariate correlations were analyzed by Spearman's correlation analysis. The results were considered statistically significant at p <0.05.

## Results

Fifty pregnant women diagnosed with gestational diabetes mellitus applied to our clinic within the indicated period. Forty-four of them met the inclusion criteria. Forty-seven pregnant women who were matched for age and pre-pregnancy BMI with the study group were identified as the control group, but 40 of them met the inclusion criteria. The demographic and clinical characteristics of the GDM and control group are presented in Table 1. Whereas there was no statistically significant difference in pre-pregnancy BMI values in both groups (p=0.111), BMI values at the time of taking maternal blood samples were significantly higher in the GDM group compared to the control group (p=0.025).

**Table 1:** The demographic and clinical characteristics of the patient with GDM and control groups. Values are given as mean ± standard deviation or median (minimum-maximum). *Mann-Whitney U-test. **Independent simple t-test. ***Chi-square- test. BMI: body mass index; GDM: gestational diabetes mellitus; Vag.delivery: vaginal delivery; C-section: cesarean section; APGAR: Appearance, Pulse, Grimace, Activity, and Respiration

		GDM (n=44)	Control (n=40)	p-Value
Maternal age (year)		31.4±3.8	30.3±4	0.083*
Gravidity		3 (1-6)	2 (1-5)	0.003*
Parity		2 (0-4)	1 (0-3)	0.035*
Prepregnancy BMI (kg/m^2^)		26.2±4,6	24,7±3,4	0.111**
BMI at sampling (kg/m^2^)		30.7± 4.9	28.5± 3.6	0.025**
Gestational age at blood sampling (days)		180±7.6	179.3±9.2	0.625*
Gestational age at delivery (days)		270.5±5	266.7±37.6	0.464*
Birth weight (g)		3245± 433	3265± 403	0.824**
Neonatal APGAR (1 min)		7.36±1.3	7.5±1.2	0.643*
Neonatal APGAR (5 min)		8.7±1.02	9.1±0.74	0.125*
Neonatal gender	Female	21 (%47,7)	22 (%55)	0.655***
	Male	23 (%52,3)	18 (%45)	
Type of delivery	Vag.delivery	28 (%63,6)	28 (%70)	0.699***
	C-section	16 (%36,4)	12 (%30)	

The biochemical values of pregnant women in GDM and control groups are shown in Table 2. It was determined that fasting plasma glucose and 50 g OGTT first-hour plasma glucose were significantly higher in the GDM group (p=0.005, p<0.001, respectively). Similarly, it was found out that HbA1c and HOMA-IR values were also higher in the GDM group compared to the control group (p=0.030, p=0.019, respectively). It was determined that maternal serum DLK1 and nesfatin-1 values were at lower concentrations in the GDM group compared to the control group (p=0.002, p<0.001, respectively).

**Table 2:** Biochemical values of the patient with GDM and control groups. Values are given as mean ± standard deviation. *Mann-Whitney U-test. **Independent simple t-test. GDM: gestational diabetes mellitus; HOMA-IR: homeostatic model assessment insulin resistance index; HDL: high-density lipoprotein; LDL: low-density lipoprotein; OGTT: oral glucose tolerance test; HbA1c: glycosylated hemoglobin; DLK1: delta-like 1; TSH: thyroid-stimulating hormone

	GDM (n=44)	Control (n=40)	p-Value
TSH (µIU/mL)	1.6±1.2	1.7±0.76	0.169*
1st hour PG 50 g OGTT (mg/dL)	180.4±28.5	120.5±21.4	<0.001**
Fasting glucose (mg/dL)	88.6±16.1	79.7±6.6	0.005*
Fasting insulin (µU/mL)	12.9±5.2	11.08±4.6	0.097*
Satiety glucose (2 h) (mg/dL)	106.3±37.2	99±16.5	0.273*
Satiety insulin (2 h) (mIU/L)	53.2±42	51.2±42	0.907*
HbA1c (%)	5.4±0.6	5.1±0.4	0.030**
HOMA-IR	2.85±1.38	2.2±1.01	0.019
HDL (mg/dL)	52.4±11.2	56.2±9.2	0.189*
LDL (mg/dL)	119.3±11.6	118.8±29.2	0.064*
Total cholesterol (mg/dL)	207.02±16.3	207.04±32.1	0.996**
Triglyceride (mg/dL)	176.1±32.8	171.2±38.5	0.911*
Maternal serum nesfatin-1 (ng/mL)	12.2±7.6	26.7±16.4	<0.001*
Maternal serum DLK1 (ng/L)	418.4±282.6	586.7±303	0.002*

Spearman's correlation analysis of nesfatin-1 and DLK1 with biochemical and anthropometric measurements is shown in Table 3. A positive correlation was determined between DLK1 and HOMA-IR and fasting insulin (r=0.395, p=0.008; r=0.374, p=0.012, respectively).

**Table 3:** Spearman correlation coefficients (r) between nesfatin-1, DLK1, and measured parameters in GDM group. BMI: body mass index; GDM: gestational diabetes mellitus; HOMA-IR: homeostatic model assessment insulin resistance index; HDL: high-density lipoprotein; LDL: low-density lipoprotein; OGTT: oral glucose tolerance test; HbA1c: glycosylated hemoglobin; DLK1: delta-like 1; TSH: thyroid-stimulating hormone

Variable	DLK1		Nesfatin-1	
	r-Value	p-Value	r-Value	p-Value
Prepregnancy BMI (kg/m^2^)	0.027	0.862	0.099	0.523
BMI at sampling (kg/m^2^)	0.018	0.907	0.093	0.550
Fasting glucose (mg/dL)	0.097	0.531	0.032	0.835
1st hour PG 50 g OGTT	-0.282	0.064	-0.195	0.203
Fasting insülin (µU/mL)	0.374	0.012	0.127	0.411
HOMA-IR	0.395	0.008	0.167	0.279
HbA1C (%)	0.040	0.796	0.059	0.702
HDL (mg/dL)	0.024	0.875	-0.034	0.826
LDL (mg/dL)	-0.006	0.971	-0.131	0.396
Total cholesterol (mg/dL)	-0.084	0.587	-0.162	0.294
Triglyceride (mg/dL)	-0.161	0.296	-0.008	0.959
Birth weight (g)	-0.115	0.459	-0.181	0.240

## Discussion

Insulin resistance plays a critical role in the physiopathology of GDM. It has been demonstrated that various adipokines are irregular in GDM, yet exactly how it impacts is still uncertain. In our study, we evaluated the concentrations of DLK1 and nesfatin-1, which are closely related to insulin resistance, in pregnant women with GDM and their association with metabolic parameters. We detected that maternal serum levels of both DLK1 and nesfatin-1 were lower in the GDM group. Moreover, we found out that there is a positive relationship between maternal serum DLK1 concentrations and fasting insulin and HOMA-IR.

DLK1 was found to be higher in small-for-gestational-age (SGA) infants [20] and infants born to mothers with preeclampsia in previous studies [21]. For the first time, Li et al. also evaluated levels of DLK1 in the cord blood of mothers with GDM and determined it lower [22]. Later, Wurst et al. assessed the DLK1 level among pregnant women with GDM, but contrary to their hypothesis, they did not detect any difference in GDM compared to the control group. Besides, the negative correlation between DLK1 levels and BMI and C-reactive protein (CRP) is remarkable [23]. The fact that GDM is an inflammatory process, and women with GDM are predisposed to be more obese might corroborate the lower DLK1 in pregnant women with GDM in our study. Although Wurst et al.’s study and the method of our study are similar, our results differ. We think that this difference may arise from the study population and/or the use of different DLK1 test kits.

Zhao et al. revealed that the DNA region in the DLK1 gene promoter was hypermethylated due to hyperglycemia in mothers with GDM and ultimately, DLK1 expression in the placenta decreased significantly in pregnant women with GDM. Moreover, while the methylation status of the DLK1 gene on the maternal side of the placenta indicated a strong correlation with the maternal two-hour OGTT glucose level, the methylation status on the fetal side of the placenta showed a high correlation with fetal birth weight [24]. It has been suggested that disruption of DNA methylation due to hyperglycemia might lead to abnormal placental DLK1 expression, causing metabolic disorders like obesity and insulin resistance to be passed on to future generations [12]. However, recent evidence has shown that the unregulated expression of imprinted genes could play a role in postnatal metabolic disorders, through its effect on embryonic growth and development [11].

Albeit DLK1 is known as an inhibitor of adipocyte differentiation [25,26], there is also a study showing that it might increase adipocyte differentiation [27]. The risk of GDM increases with advanced age and obesity. Jensen et al. determined that DLK1 is associated with increased obesity. This effect might be due to a direct effect of DLK1 on adipose tissue as well as due to the fact that it increases insulin-dependent fat accumulation by increasing insulin secretion in the pancreas [11]. These data are also corroborated by the study of Hudak et al., which has shown that DLK1 ablation reduces the development of white adipose tissue (WAT) significantly [28]. Yet, these findings contradict studies, which have demonstrated that DLK1-null mice are more obese [25] and that DLK1-overexpressing mice are leaner [26]. This contradiction is mostly attributed to the difference in body fat measurement techniques used in the studies [11]. However, we did not find any correlation between BMI and DLK1 concentrations in our study. This might be due to the difference in BMI of our study population at the time of sampling in both groups.

Findings that both DLK1-null and DLK1-overexpressing transgenic mice develop insulin resistance and glucose intolerance also indicate that proper development of adipose tissue function might be critical for maintaining glucose/insulin homeostasis [25,26]. It is well-known that DLK1 has a negative impact on body glucose homeostasis through inhibiting glucose transporter type 4 (GLUT4)-mediated glucose uptake in skeletal muscle [11]. Likewise, we also considered that GDM, which is based on insulin resistance, might be associated with DLK1, and we determined a positive correlation between DLK1 levels and HOMA-IR among patients with GDM. Whereas this finding is similar to the findings of Lee et al.’s study [26], it contradicts Charalambous et al.’s study [27]. This could be due to differences in the study population and/or the use of different assays for DLK1 quantification. Ultimately, more detailed studies are needed in vivo and in vitro to unveil the relationship of DLK1 with insulin resistance.

In addition to having an insulinotropic effect due to glucose, nesfatin-1 also plays a role in carbohydrate metabolism by inhibiting glucagon secretion [16]. Studies on nesfatin-1 levels in GDM have been performed, but the results have been found to be contradictory. Even, Mierzyński et al. used ELISA kits of different companies, and similar to the results of our study, they found lower nesfatin-1 levels in pregnant women with GDM compared to healthy pregnant women [1]. Insulin resistance and hyperinsulinemia play a role in the pathophysiology of both GDM and T2DM, and both are more common among overweight and obese individuals. Results of the studies that assess nesfatin-1 levels in T2DM are also uncertain, as in women with GDM. We found in our study that nesfatin-1 concentrations were at lower concentrations in pregnant women with GDM. This observation might indicate that nesfatin-1 synthesis or secretion is impaired in women with GDM and might have a role in GDM development. Aslan et al. showed that nesfatin-1 values in both maternal and cord blood were lower in women with GDM, which supports our study [14]. Contrary to this, Zhang et al. detected higher nesfatin-1 concentrations among pregnant women with GDM [17]. It has been revealed in a meta-analysis that these conflicting results might be due to the differences in the geographical region and the applied diagnostic criteria. For instance, whereas nesfatin-1 was detected at lower levels in Caucasian pregnant women with GDM, it was found similar to healthy pregnant women in Asian pregnant women with GDM [29]. Moreover, whereas the concentrations of nesfatin-1 in the circulation in women diagnosed with GDM according to Coustan and Carpenter (C&C) and WHO criteria were lower than controls, it was determined higher in pregnant women with GDM diagnosed in accordance with the International Association of Diabetes and Pregnancy Study Groups (IADPSG) criteria compared to the control group [29]. Nesfatin-1, which is directly related to appetite, might play a role in regulating body weight in pregnant women, albeit the effects of nesfatin-1 on the underlying pathogenesis of GDM are not entirely figured out. Given that individuals with high body weight are predisposed to GDM, it is an expected result that nesfatin-1 concentrations are found to be lower among pregnant women with GDM.

The robustness of our study is that we have assessed two adipokines, levels of which in GDM are still uncertain. The limitations of our study are that our sample group is relatively small, we only evaluated maternal blood samples, and there are no long-term neonatal results.

## Conclusions

Today, the only method for screening GDM is OGTT. However, some pregnant women avoid this method because they think that drinking sugar water is harmful to their baby or because they do not want to give blood samples several times. In this case, there is a need for alternative methods to OGTT. In conclusion, we found lower DLK1 and nesfatin-1 concentrations among pregnant women with GDM compared to healthy pregnant women. If these results can be verified by further studies, DLK1 and nesfatin-1 levels can be a new marker to determine women with GDM.
